# Repositioning of Etravirine as a Potential CK1ε Inhibitor by Virtual Screening

**DOI:** 10.3390/ph15010008

**Published:** 2021-12-22

**Authors:** Luis Córdova-Bahena, Axel A. Sánchez-Álvarez, Angel J. Ruiz-Moreno, Marco A. Velasco-Velázquez

**Affiliations:** 1 Departamento de Farmacología, Facultad de Medicina, Universidad Nacional Autónoma de México, Mexico City 04510, Mexico; luisbahena@unam.mx (L.C.-B.); aaalvarez@comunidad.unam.mx (A.A.S.-Á.); angel.j.ruiz.moreno@gmail.com (A.J.R.-M.); 2 Unidad Periférica de Investigación en Biomedicina Traslacional CMN 20 de noviembre ISSSTE, Facultad de Medicina, Universidad Nacional Autónoma de México, Mexico City 04510, Mexico; 3 Consejo Nacional de Ciencia y Tecnología (CONACYT), Mexico City 03940, Mexico; 4 Facultad de Química, Universidad Nacional Autónoma de México, Mexico City 04510, Mexico; 5 Programa de Doctorado en Ciencias Biomédicas, Universidad Nacional Autónoma de México, Mexico City 04510, Mexico

**Keywords:** cancer, drug repurposing, pharmacophore model, CK1ε, etravirine, abacavir

## Abstract

CK1ε is a key regulator of WNT/β-catenin and other pathways that are linked to tumor progression; thus, CK1ε is considered a target for the development of antineoplastic therapies. In this study, we performed a virtual screening to search for potential CK1ε inhibitors. First, we characterized the dynamic noncovalent interactions profiles for a set of reported CK1ε inhibitors to generate a pharmacophore model, which was used to identify new potential inhibitors among FDA-approved drugs. We found that etravirine and abacavir, two drugs that are approved for HIV infections, can be repurposed as CK1ε inhibitors. The interaction of these drugs with CK1ε was further examined by molecular docking and molecular dynamics. Etravirine and abacavir formed stable complexes with the target, emulating the binding behavior of known inhibitors. However, only etravirine showed high theoretical binding affinity to CK1ε. Our findings provide a new pharmacophore for targeting CK1ε and implicate etravirine as a CK1ε inhibitor and antineoplastic agent.

## 1. Introduction

The casein kinase 1 (CK1) family comprises enzymes that regulate signal transduction pathways by reversible phosphorylation of their substrate proteins [1]. They are involved in many cellular processes, including DNA repair, cell differentiation, intracellular trafficking, immune responses, and apoptosis [2]. In mammals, the CK1 family has seven members: α, β1, γ1, γ2, γ3, δ, and ε.

Physiologically, CK1ε participates in circadian clock control via phosphorylation of PER2 and PER3. Phosphorylated PER2/3 translocate into the nucleus, suppressing the activity of the CLOCK/BMAL1 transcriptional complex [3]. However, CK1ε also modulates the transduction of many signals in cancer cells. For example, it phosphorylates p53 and Mdm2, which are important in cell proliferation and the maintenance of genomic integrity [4,5].

Further, CK1ε is a pivotal regulator of the WNT pathways [6], which are commonly altered in various human cancers [7,8]. The upregulation of CK1ε activity that is elicited by WNT ligands [9] leads to the phosphorylation of Dishevelled [10], activating the canonical WNT pathway. Conversely, in the absence of WNT ligands, CK1ε phosphorylates β-catenin, promoting its degradation [11]. This latter activity occurs in glioblastoma cells, in which the of inhibition CK1ε activates β-catenin and induces apoptosis [12]. CK1ε can also interact and phosphorylate the tyrosine-protein kinase WNT co-receptors ROR1/ROR2 in cancer cells [13,14], triggering AKT-mediated signaling and promoting proliferation [14]. Furthermore, CK1ε protects ROR2 protein from degradation [15].

Addition protumoral effects of CK1ε include its control of the expression of the mitochondrial protein adenine nucleotide translocase 2 (ANT2). In ovarian cancer cells, CK1ε interacts with ANT2 to support ATP production [16]. Accordingly, the inhibition of CK1ε suppresses cell proliferation, reduces xenograft growth in vivo, and increases the susceptibility to chemotherapy. Thus, CK1ε is considered an antineoplastic target on several levels [17,18,19].

Structurally, CK1ε has a highly conserved kinase domain that is organized into a bilobal arrangement [20,21]. The N-terminal lobe is composed primarily of β-sheets, and the C-terminal lobe comprises α-helices [22]. The catalytic site is located between the two lobes. An analysis of the binding mode of ATP has defined five regions [23]: (1) adenine-interacting, (2) sugar-interacting, (3) phosphate-interacting, (4) buried region, and (5) solvent accessible region. The active residues for ATP binding are Ala36, Lys38, and Met80 in the N-lobe; Glu90, Leu91, and Phe95 in the C-lobe; and Met82, Glu83, Leu84, and Leu85 in the linker loop between the two lobes [24].

This extensive characterization of the catalytic pocket of CK1ε has guided the development of several competitive inhibitors with moderate to high biological activity [24,25,26,27]. For example, the CK1ε inhibitor PF-4800567 (hereafter referred to as inhibitor 1 [IN1]) is an efficacious inhibitor of circadian rhythms in cycling Rat1 fibroblasts and mice [27]. In February 2021, the Food and Drug Administration (FDA) granted approval to the CK1ε inhibitor umbralisib for the treatment of marginal zone lymphoma and follicular lymphoma [28]. Nevertheless, no other CK1ε inhibitor has attained clinical use, highlighting the need of new inhibitors.

We generated a pharmacophore model by characterizing the binding modes of five reported CK1ε inhibitors. Our model was then used to identify potential molecules that bind the catalytic domain of CK1ε from a database of FDA-approved drugs. We found that the anti-HIV drugs etravirine and abacavir have conformers that match our pharmacophore and were the most likely binding modes in molecular docking experiments. Etravirine emulated the noncovalent interactions in reported CK1ε inhibitors, with a similar theoretical ∆G as IN1. Further, we observed that additional residues outside of the catalytic domain participated in stabilization of the etravirine-CK1ε interaction. Thus, we propose the repurposing of etravirine as a CK1ε inhibitor. Biological validation of our findings will constitute the basis for the development of new clinical CK1ε inhibitors.

## 2. Results

### 2.1. Identification of Binding Modes for Noncrystallized CK1ε Inhibitors

**Figure 1:**
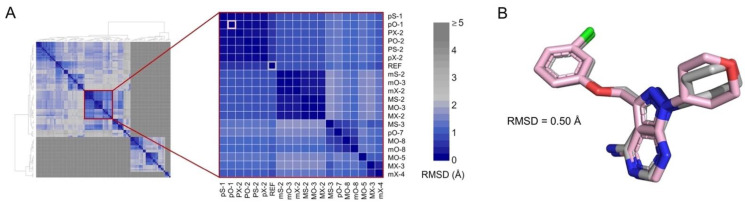
Docking protocol reproduces the active conformer of IN1. (**A**) Hierarchical clustering of 96 poses obtained by molecular docking. Inset shows the largest subcluster. Poses are labeled using letters that indicate the combination of search algorithm/scoring function employed (see “Methods”) and a number indicating the ranked position. The crystal pose of IN1 (REF) and the best scored pose calculated using PLANTS scoring function and Optimizer search algorithm (pO-1) are highlighted in gray and pink, respectively. Color scale shows the RMSD between poses. (**B**) Superimposition of co-crystallized (gray) and pO-1 docked (pink) poses.

The set of inhibitors used in this study is listed in Table 1. The binding mode for inhibitors without available structural data (inhibitors [IN] 2–5) was determined by molecular docking. The protocol was validated by docking IN1 into the catalytic site of CK1ε using 12 combinations of search algorithms and scoring functions. Eight poses were calculated for each combination. Hierarchical clustering of the 96 predicted poses identified subclusters with poses that had a root-mean-square deviation (RMSD) of atomic positions <2.0 Å (Figure 1A). A comparison of a representative pose of the largest subcluster and the bioactive conformer in the structure [22] of PDB 4HNI yielded an RMSD of 0.5 Å (Figure 1B). The largest subcluster included the binding modes with the best scores for various combinations of search algorithms and scoring functions, indicating that the strategy can predict the experimental binding mode.

**Figure 2:**
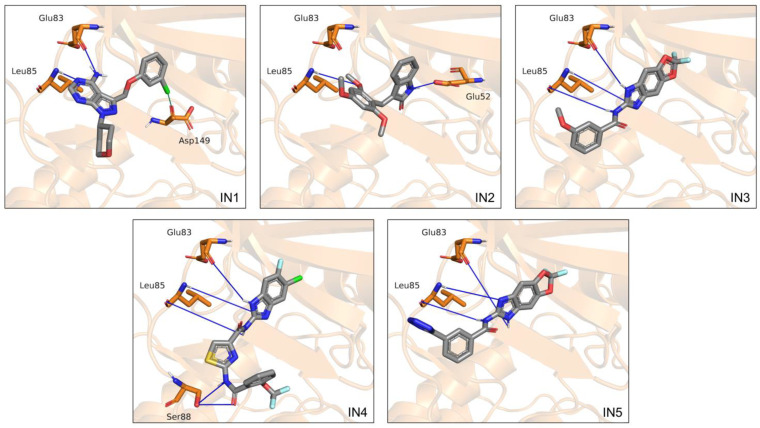
Binding modes calculated by molecular docking for the five inhibitors analyzed. Cartoon representation of CK1ε with residues Glu52, Glu83, and Leu85, and Ser88 on licorice model. Hydrogen bonds are shown as blue lines.

Four additional CK1ε inhibitors with unknown binding mode were docked using the validated protocol. For these inhibitors, the largest subclusters that were generated by hierarchical clustering included poses with RMSD < 2.0 Å (Figure S1). From the representative poses of these subclusters, we identified the binding regions and the intermolecular interactions formed (Figure 2). For all inhibitors, the binding modes were mainly driven by hydrogen bonds with the backbone of residues Glu83, and Leu85. Additional hydrogen bonds were found with Glu52 and Ser88, for IN2 and IN4 respectively. The fused rings of IN2, IN3, IN4, and IN5 were oriented toward the buried region of the catalytic pocket, and the groups on the side opposite to the fused rings were oriented to the solvent accessible region. In contrast, the fused rings of IN1 remained directed toward the adenine region in the middle area of the catalytic pocket. In addition, for all inhibitors the binding was mediated largely by hydrophobic interactions, although charged amino acids also participated.

### 2.2. Characterization of Relevant CK1ε-Inhibitor Interactions by Molecular Dynamics

To examine the conformational dynamics of the ligands in the catalytic site of CK1ε, we performed simulations by molecular dynamics (MD). As expected for compounds with proven biological activity, all systems showed stable binding, although IN1 and IN2 conformers had greater stability (average RMSD < 2 Å) than IN3 and IN5 (average RMSD < 4 Å). The greatest changes for IN3 and IN5 occurred in the anisole and tetrazole groups, respectively, in the solvent accessible region. For IN4, the ligand showed transient variability at the beginning of MD but eventually stabilized.

**Figure 3:**
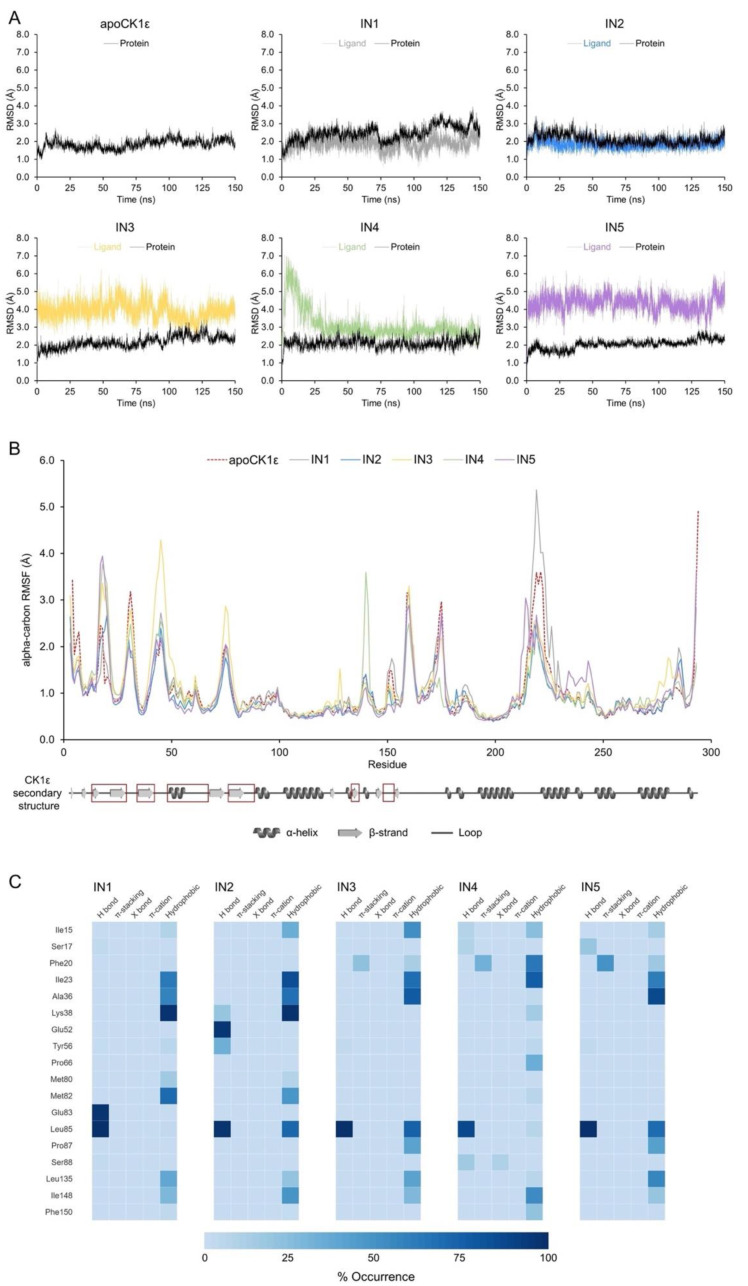
Molecular dynamics analysis identified key inhibitor-CK1ε interactions. (**A**) Protein backbone and ligand RMSDs for apoCK1ε and CK1ε-inhibitor complexes. (**B**) Protein backbone RMSF for all systems. Secondary structure of CK1ε is shown at the bottom with regions comprising the catalytic domain in red squares. (**C**) Heatmap of the non-covalent interaction profile occurrence. For clarity, only residues with occurrence higher than 20% are shown.

RMSDs of the protein backbone in the apo enzyme and CK1ε-inhibitor complexes were used to analyze structural changes in the target protein. The complete trajectory for the apo enzyme showed an RMSD value of 1.85 ± 0.26 Å (average ± standard deviation). For the CK1ε-inhibitor complexes, the average RMSD was below 2.5 Å, indicating that no significant structural changes occur in CK1ε when bound to inhibitors (Figure 3A).

The root-mean-square fluctuation (RMSF) values from atomic positions of the alpha carbons in the backbone protein showed that all systems behaved similarly, except for a few residues. The loops that comprised residues 42–48 and 74–76 were particularly flexible in the complex with IN3 compared with other inhibitors. Similarly, the 139–140 loop and 216–226 loop-helix, which lie outside of the catalytic site, showed increased mobility in the complexes with IN4 and IN1, respectively (Figure 3B).

An analysis of the 15,000 bioactive conformations that were accessible for each inhibitor allowed us to create dynamic noncovalent interaction profiles for each residue (Figure 3C). Hydrogen bonds with the Leu85backbone in the adenine region were noted, ranging from 13,204 to 14,998 conformations (88.02% to 99.99% occurrence) for all inhibitors, indicating that such an interaction occurs independently of the inhibitor. Similar but less frequent interactions were identified for residues Ile15, Ile23, Leu135, and Ile148 for all inhibitors. Conversely, Phe20 in the phosphate-binding region of the catalytic domain formed 3239 pi-stacking interactions in the simulation for IN3, 4713 for IN4, and 7421 for IN5 (occurrence of 21.59%, 31.42%, and 49.47%, respectively); thus, pi-stacking that is mediated by Phe20 favors the binding of certain compounds.

Finally, we identified residues that mediate only the binding of a particular inhibitor. Hydrophobic interactions with Phe20 and Pro66 occurred frequently for IN4 (63.36% and 33.79% of the time, respectively). Hydrogen bonds with Glu83 appeared for IN1 (97.48% of the time). Several hydrogen bonds with Lys38 and Glu52 were found with IN2; however, Lys38 participated in binding primarily through hydrophobic interactions, and Glu52 had no function in any of the other inhibitors.

### 2.3. Generation of Pharmacophore Model

**Figure 4:**
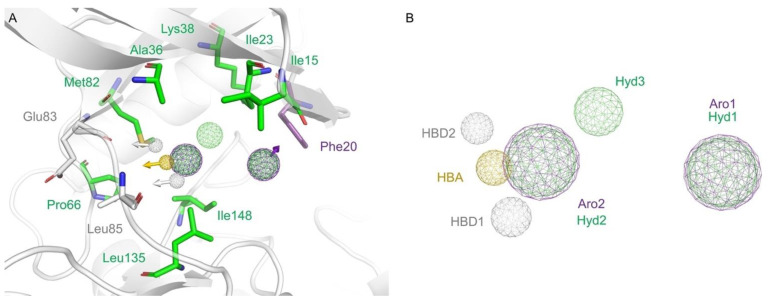
Pharmacophore modeling. (**A**) CK1ε catalytic site representation with selected amino acids showed on licorice model. Residues forming hydrophobic interactions, hydrogen bonds, or stacking interactions are colored green, yellow and white, or purple, respectively. (**B**) The generated pharmacophore model included eight elements. Hydrogen bond donors (HBD) are represented as sphere grid colored white, hydrogen bond acceptor (HBA) on yellow, aromatic (Aro) on purple, and hydrophobic (Hyd) on green.

Based on their frequency in our MD analyses, we selected eight intermolecular interactions to generate a 3D pharmacophore model (Figure 4):

1. a hydrogen bond acceptor (HBA) from the interactions of all inhibitors with the Leu85 backbone;
2. a hydrogen bond donor (HBD1) from the interactions of all inhibitors with the Leu85 backbone;
3. a second hydrogen bond donor (HBD2) from the interaction of IN1 with Glu83;
4. an aromatic (Aro1) feature from the pi-stacking interactions of IN3, IN4, and IN5 with Phe20;
5. a hydrophobic (Hyd1) feature—at the same position of Aro1—from the hydrophobic interaction of IN4 with Phe20;
6. a second hydrophobic element (Hyd2) from the interactions of inhibitors with Ala36, Pro66, Met82, Leu135, and Ile148;
7. a second aromatic element (Aro2)—at the same position as Hyd2—because a ring can fix the hydrogen bond elements to each other;
8. a third hydrophobic element (Hyd3) from the interactions of IN1 and IN2 with the lateral chain of Lys38.

### 2.4. Virtual Screening

The generated pharmacophore was used to search a virtual library of FDA-approved drugs. When the complete pharmacophore model was queried, only etravirine appeared as a match. Thus, we performed a second search with a submodel without the Aro1/Hyd1 dual element. These elements were eliminated because Hyd1 makes a less important energetic contribution and because the position of the element remains solvent-exposed. Seven additional compounds matched this simplified pharmacophore model. The set of candidate drugs is presented on Table S1.

**Figure 5:**
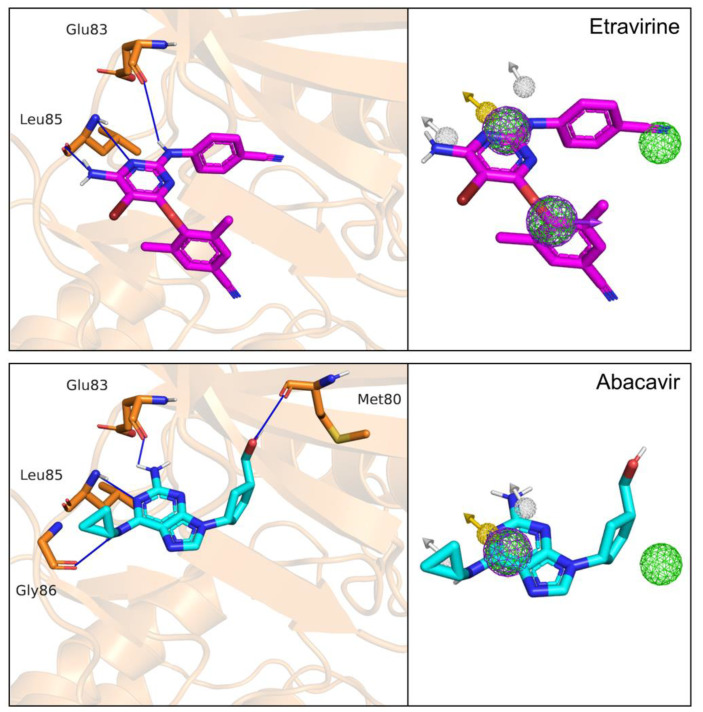
Docking poses of etravirine and abacavir. Overlap of pharmacophore features with the calculated binding modes of etravirine (magenta) and abacavir (cyan). Hydrogen bonds are shown as blue lines. Note that the pharmacophoric submodel used for abacavir lacks the dual element Aro1/Hyd1 (see text for details).

Of the eight identified drugs, four were discarded because they have predominant species at physiological pH with protonation states different to the species matching the pharmacophore. The remaining four compounds were docked into the catalytic site of CK1ε to analyze their binding mode. Two compounds bound to the target in modes that clearly differed from that predicted by the pharmacophore models (RMSD > 5.1 Å) and thus were not further studied. In contrast, etravirine and abacavir bound to CK1ε per the model that was used for their identification (Figure 5).

### 2.5. MD Analyses Support the Repurposing of Etravirine as a CK1ε Inhibitor

**Figure 6:**
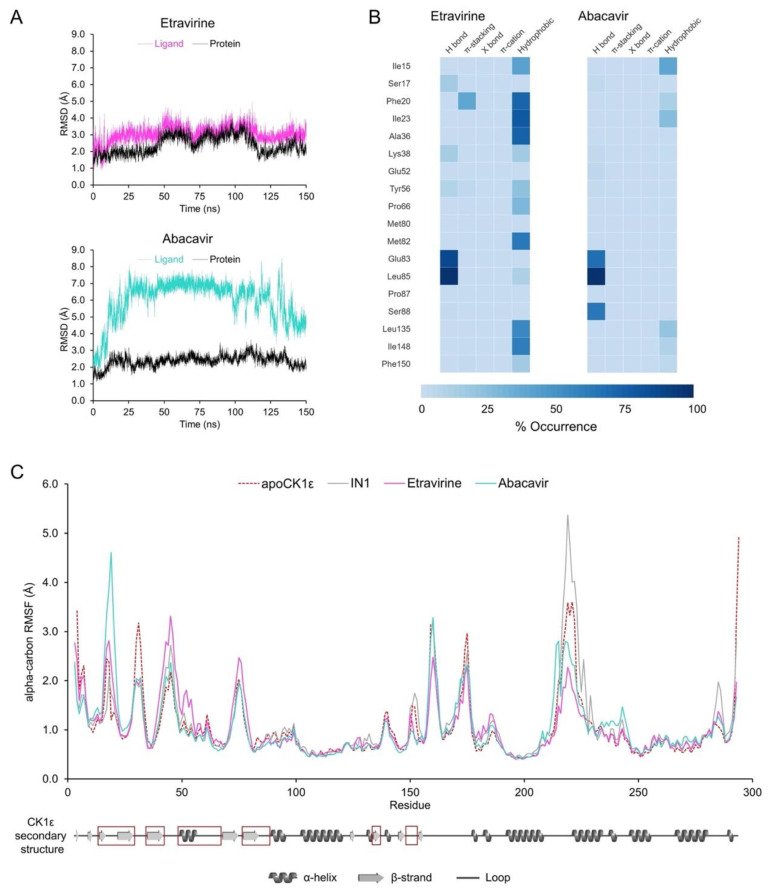
MD analyses for CK1ε-etravirine and CK1ε-abacavir complexes. (**A**) Protein backbone (black) and ligands (colored) RMSDs. (**B**) Heatmap of the non-covalent interactions occurrence for selected residues. (**C**) Protein backbone RMSF. The apo enzyme and the system CK1ε-IN1 are shown for comparison. Secondary structure of CK1ε is shown at the bottom with the catalytic domain in red squares.

Complexes of etravirine or abacavir with CK1ε were examined by MD. The CK1ε-etravirine complex had an RMSD of 2.4 ± 0.5 Å for the enzyme backbone and 3.1 ± 0.5 Å for the ligand, suggesting that the system remains stable during the MD simulation (Figure 6A). Further, etravirine reproduced the pattern of interactions in the known inhibitors (Figure 6B). Etravirine formed a trident of hydrogen bonds with Glu83 and Leu85, with a prevalence of 87.7% and 98.3%, respectively, playing a major role in binding of the compound. Similarly, stacking interactions with Phe20 were 38.2% of the time. In addition, several hydrogen bonds were noted with Ser17, Lys38, and Tyr56 13.7%, 13.1%, and 7.8% of the time, respectively, but hydrophobic interactions were predominant.

The CK1ε-abacavir complex had an RMSD of 2.4 ± 0.3 Å for the enzyme backbone and 6.0 ± 1.5 Å for the ligand (Figure 6A), indicating that although the protein remained stable, abacavir undergoes significant conformational changes. A dynamic noncovalent interaction profile analysis (Figure 6B) identified the predicted hydrogen bonds with Glu83 and Leu85 at a prevalence of 67.7% and 98.8%, respectively, and an additional one with Ser88 at 63.4%. Thus, abacavir partially reproduces the interactions in the pharmacophore, lacking stacking interactions and participating in fewer hydrophobic interactions.

The RMSF analysis (Figure 6C) showed that fluctuation in Ser31, which lies adjacent to the catalytic domain, is reduced by both drugs, compared with the apo enzyme. Similar behavior occurred with the control compound IN1. The CK1ε-abacavir complex showed high fluctuation in Phe20 versus systems with IN1 or etravirine, in which Phe20 was rigid due to stacking interactions with the ligand. Finally, the binding of both drugs restricted the conformational dynamics of the loop conformed by residues 217–226. Given that such a loop showed increased fluctuation with IN1 and it resides outside of the catalytic site, the relevance of the changes that are induced by etravirine and abacavir remain to be determined.

**Figure 7:**
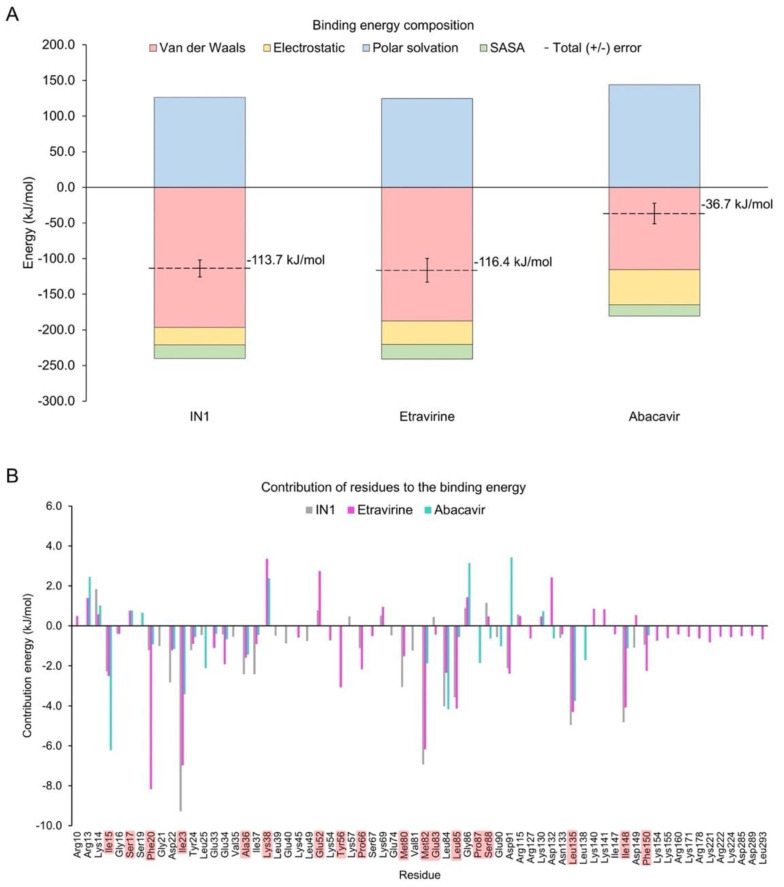
Binding energy-analysis. (**A**) Energy decomposition from systems with etravirine or abacavir were calculated from MD simulations. The system with IN1 is included for comparison. (**B**) Per-residue energy decomposition. Highlighted residues are part of the catalytic domain.

The binding energy of the identified drugs was calculated by MM-PBSA for the entire MD simulation (Figure 7A). The contribution of van der Waals interactions and energy that was associated with a solvent-accessible surface were similar in magnitude for etravirine and IN1, but the electrostatic contribution was slightly higher for etravirine. Thus, the total binding energy of etravirine to CK1ε approximates that of IN1. Conversely, the total binding energy for abacavir was lower by three-fold, suggesting that this drug should not be prioritized in experimental assays.

Analysis of the energetic contribution of each residue to the binding energy (Figure 7B) showed that residues Asp22, Ile23, Tyr24, Ala36, Met82, Leu135, and Ile148 contributed more to IN1 but still cooperated in the binding of etravirine and abacavir. Phe20 was crucial in binding etravirine but was minor in interactions with abacavir and IN1. Similar behavior was observed for Glu34, Lys45, Lys54, Tyr56, Pro66, Leu85, and Phe150. Notably, the large and positively charged residues Lys155, Lys171, Arg178, Lys221, Arg222, and Lys224, located in front of the catalytic domain, contributed uniquely to the binding mode of etravirine. However, Lys38, Glu52, Lys69, Gly86, Arg115, Lys130, Asp132, Lys140, Lys141, and Asp149 negatively affected the stability of the CK1ε-etravirine complex. To determine the function of Lys38 and Glu52, the prevalence of a salt bridge between such residues in the CK1ε-etravirine complex was evaluated. Our findings suggest that etravirine competes with Glu52 for Lys38 (Figure S2).

## 3. Discussion

CK1ε has been implicated as a biological target due its importance in the initiation and progression of various types of cancer [1,4,5,6,12,17,29]. In this study, we aimed to identify potential CK1ε inhibitors by repurposing FDA-approved drugs by virtual screening. Drug repurposing is an effective strategy for identifying new activities of approved drugs [30,31,32]. This approach has several advantages, including its accelerated clinical translation, given the known pharmacokinetics and safety profiles of the candidate compounds [33].

Although several ATP-competitive inhibitors of CK1ε have been reported [22,24,25,26], only 1 has a known binding mode [22]. Thus, we determined the most likely binding modes of four additional CK1ε inhibitors, observing that a fraction of each molecule remains solvent-exposed in the limits of the catalytic pocket, as has been reported for IN1 [22]. The binding of ligands is mediated primarily by hydrophobic residues into the catalytic site. The inhibitors form nonpolar interactions with the side chains of Ile23 and Ala36 and the aliphatic chain of Lys38, located in the buried region of the catalytic pocket. In addition, the backbone of Leu85 forms hydrogen bonds with all inhibitors, as has been reported for the adenine moiety in the binding mode of ATP [20]. These results are in good agreement with the reported binding modes of CK1δ inhibitors and other inhibitors of kinases with bilobal stucture [34,35,36]. Our findings suggest that common key residues of CK1ε mediate the binding of various inhibitors.

To generate information on the prevalence of such interactions, we performed MD experiments for all five inhibitors. As suggested by our previous experiments, the backbone of Leu85 plays a major role in the binding of the inhibitors, forming hydrogen bonds most of the time. Further, the Leu85 and Glu83 backbones remain highly synchronized, making hydrogen bonds with IN1. We also identified new, frequent hydrophobic interactions with Met82, Leu135, and Ile148, in addition to those by Ile23, Ala36, and Lys38. Finally, Phe20, in the phosphate-binding region, forms stacking interactions that stabilize the regions of the inhibitors that remain exposed to the solvent.

These identified patterns of intermolecular interactions drove the generation of a target-based pharmacophore. A similar strategy has been used successfully in the identification of kinase inhibitors [37,38,39]. Our model is disposed on a triangle conformation, wherein the first one of the vertexes is situated in the buried region, the second one is situated interacting with the adenine region, and the third one is situated at the phosphate binding region. This model is compatible with the one proposed by Bolcato et al. for CK1δ inhibitors [40], but implies improvement since our analysis revealed additional hotspots.

The pharmacophore, and a simplified submodel in which we removed the stacking interaction with the phosphate-binding region, allowed us to identify the non-nucleoside reverse-transcriptase inhibitors etravirine and abacavir from a library of approved drugs. The pharmacophore that we have described can also be used to search for hits in other chemotheques to identify additional compounds that can be developed into CK1ε inhibitors. Given that our study aimed to repurpose approved drugs, additional screens are outside of its scope, constituting a limitation of this report.

Our MD analysis showed that both drugs stably bind CK1ε, reproducing the interactions that conform the pharmacophore(s). Although both compounds remained fixed to the interconnection loop between lobes, important differences between their binding modes were noted. For etravirine, we corroborated the key role of Phe20. There was also cooperation of additional hydrogen bonds between the ammonium group of Lys38 and the nitrile substituent of the benzonitrile moiety. However, in apo CK1ε, Lys38 and Glu52 frequently form a salt bridge, but etravirine competes with Glu52 for Lys38, driving the energetic contribution of Lys38 to become unfavorable. Conversely, additional hydrogen bonds that formed between the benzonitrile moiety of etravirine and Tyr56 were beneficial for the binding mode. Further, the aliphatic residues Ile15, Ile23, Ala36, Pro66, Leu85, Pro87, Leu135, Ile148, and Phe150 contributed favorably to the binding mode. Notably, many charged residues in the C-lobe contribute favorably to the binding energy, except for Glu52 and Asp132, which interacted with the two nitrile substituents of etravirine, having a prejudicial effect. Finally, the theoretical binding affinity of etravirine approximated that of the reference compound IN1.

In contrast, the calculated binding affinity of abacavir to CK1ε was significantly reduced. As expected for a hit that was obtained with a pharmacophore that lacked the stacking interaction with Phe20, this residue was irrelevant to the interaction. However, we identified several hydrogen bonds with Glu83, Leu85, and Ser88; thus, these three residues fix the fused rings of abacavir. Yet, the cyclopropane moiety remained in movement without strong interactions. In contrast to etravirine and IN1, abacavir received little contribution from van der Waals interactions. Hydrogen bonds appeared in the adenine region, pulling the compound from the pocket and preventing the interaction between the hydroxymethyl cyclopentene and the buried region. Thus, abacavir is not a promising candidate for inhibiting CK1ε.

In summary, our results support the repurposing of etravirine as a CK1ε inhibitor and antineoplastic agent. Notably, etravirine activates the WNT pathway in osteosarcoma cells, increasing the expression of the cyclin-dependent kinase (CDK) inhibitor p21 [41]. This effect correlates with our findings and encourages further studies. Our data suggest that etravirine inhibits CK1ε at similar concentrations as IN1 [22,27].

## 4. Materials and Methods

### 4.1. Protein Preparation

The structures of CK1ε apo (PDB ID 4HOK), alone and cocrystallized with IN1 (PDB ID 4HNI) [22], were obtained from the RCSB Protein Data Bank. The unresolved fragments of the structures were built by homology modeling using the GapRepairer server [42].

### 4.2. Selection of Inhibitors and Ligand Preparation

Five potent CK1ε inhibitors were selected for this study. The ligand 3-[(3-chlorophenoxy)methyl]-1(oxan-4-yl)-1H-pyrazolo[3,4-d]pyrimidin-4-amine (IN1), was considered the reference compound. The compounds (3Z)-3-[(2,4,6-trimethoxyphenyl) methylidene]-2,3-dihydro-1H-indol-2-one (IN2), N-(2,2-difluoro-5H-[1,3]dioxolo[4,5-f]benzimidazol-6-yl)-3-methoxybenzamide (IN3), N-(5-chloro-6-fluoro-1,3-benzodiazol-2-yl)-4-[2-(trifluoromethoxy)benzamido]-4,5-dihydro-1,3-thiazole-2-carboxamide (IN4), and N-(2,2-difluoro-5H-[1,3]dioxolo[4,5-f]benzimidazol-6-yl)-3-(2H-tetrazol-5-yl)benzamide (IN5) were selected because they have reported IC50 values that ranged between 16–1000 nM [24,25,26].

The two-dimensional chemical structures of the ligands were drawn manually using Marvin Sketch ChemAxon, and the protonation states were calculated at pH > 7.2 (the reported intracellular pH in cancer cells) [43]. Subsequently, three-dimensional structures were built, and their geometries were optimized at the PM6 semiempirical level [44] using Spartan software, and the output files were exported in pdb format.

### 4.3. Molecular Docking

Docking simulations were performed with the Molegro Virtual Docker suite [45]. The exploration region was delimited by a 10-Å-radius sphere that was centered on the catalytic site of CK1ε, with 0.2 Å grid spacing. All rotatable bonds of ligands were set free in the experiments, and protonation states were adjusted as discussed. We used 12 combinations of 3 search algorithms [Iterated Simplex (X), MolDock Simplex Evolution (S), and MolDock Optimizer (O)] and four scoring functions [MolDock Score (m), MolDock Score [GRID] (M), Plants Score (p), and Plants Score [GRID] (P)]. For every combination, eight runs were performed, with a maximum of 1500 iterations and an initial population of 50 poses. To find the most probable binding mode, all poses were analyzed by clustering, as reported [46]. A representative pose of the largest subcluster was selected as the input file for MD simulations.

### 4.4. MD Simulations

MD simulations of the CK1ε–IN complexes were performed in GROMACS 4.5.5 [47] using the CHARMM36m force field [48]. The ligand parameters were generated with the CHARMM graphical user interface module in the force field framework [49]. Each complex was in a periodic 75 Å × 75 Å × 75 Å cubic box and solvated using a three-point (TIP3P) model for water molecules. Further, Na+ and Cl− atoms were added to neutralize the charge of the system and achieve an ionic concentration of 0.15 M. All simulations were carried out at 1 bar and 310.15 K. Before production of MD, the energy of the system was minimized using the steepest-descent algorithm, followed by equilibration in an NVT ensemble using a modified Berendsen thermostat. MD simulations were generated for 150 ns with an integration time frame of 2 fs, and the trajectories were saved after every 10 ps. Analysis of ligand-target interactions was carried out by a python tailored-made script (https://github.com/AngelRuizMoreno/Scripts_Notebooks/blob/master/Scripts/plipMD_V3.1.py; last accessed 12 December 2021) implementing MDAnalysis [50] and PLIP [51]. The complete trajectories were visualized in VMD [52].

### 4.5. Pharmacophore Modeling

We calculated the occurrence of intermolecular interactions between INs and CK1ε. We considered an interaction to be relevant only if it occurred > 20% of the total simulation time; thus, only interactions with higher frequency were analyzed to define the pharmacophoric elements. The generated pharmacophore comprised eight elements (see Results).

### 4.6. Virtual Screening

#### 4.6.1. Pharmacophore-Based Selection

Pharmacophore-matching compounds were selected from a database of 21,850 conformers from 1856 FDA-approved drugs using Pharmit [53]. Compounds with a molecular volume that was greater than 270 Å^3^ were eliminated, due to the size restrictions of the catalytic site of CK1ε. Compounds with at least six elements of the pharmacophore model were candidates for further study.

#### 4.6.2. In Silico Molecular Docking

The candidates were docked using the protocol above. Only compounds for which the most likely binding mode did not differ from the pharmacophore-predicted binding mode (RMSD < 1.5 Å) were selected.

#### 4.6.3. Binding Free Energy

The binding free energy for the etravirine-, abacavir-, and IN1-CK1ε complexes was evaluated using the complete trajectories of the MD simulations (generated as described above). We used the Poisson–Boltzmann surface area (MM/PBSA) method [54] in the g_mmpbsa v1.6 package [55]. van der Walls, electrostatic, polar solvation, and solvent-accessible surface area energies were determined to calculate the average binding energy. Per-residue decomposition analysis was performed to demonstrate the primary amino acids that were involved in stabilizing the systems.

## 5. Conclusions

In this study, two drugs that have been approved by the FDA for the treatment of HIV were identified as potential ATP-competitive CK1ε inhibitors. Both emulate the binding mode of known CK1ε inhibitors, but etravirine binds to CK1ε with greater stability and affinity. Our data encourage the evaluation of etravirine as an antineoplastic agent against CK1ε.

## Figures and Tables

**Table 1:** Characteristics of the CK1ε ATP-competitive inhibitors employed for pharmacophore modeling.

Assigned Code	Original Name	Structure	IC50 (µM)	Reference
IN1	PF-4800567	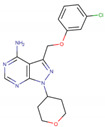	0.034 ± 0.009 *^a^*	[27]
IN2	IC261	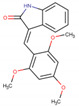	1.0 ± 0.4 *^c^*	[25]
IN3	compound No. 2	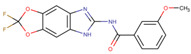	0.52 ± 0.05 *^b^* 0.16 ± 0.06 *^c^*	[26]
IN4	compound No. 6	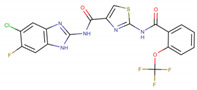	0.033 ± 0.01 *^b^*	[24]
IN5	compound No. 9	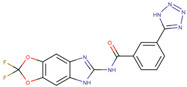	0.62 ± 0.01 *^b^*	[26]

IC50s were obtained using peptide PLSRTLpSVASLPGL *^a^*, GST-p53 *^b^*, or α-Casein *^c^* as substrates.

## Data Availability

Analyzed data are contained in the main text of the article. Raw data are available from the authors upon request.

## Supplementary Materials

The following are available online at https://www.mdpi.com/article/10.3390/ph15010008/s1, Figure S1: Docking protocol for selected inhibitors set; Figure S2: Salt bridge between Lys38 and Glu52 in CK1ε-Etravirine; Table S1: Hits from virtual screening.
