# Supplementary material for: Repositioning of Etravirine as a Potential CK1ε Inhibitor by Virtual Screening
Source: Pharmaceuticals (Basel). 2021 Dec 22;15(1):8. doi: 10.3390/ph15010008 (PMC8778358; doi:10.3390/ph15010008)
Supplement: Supplementary file 1 [file pharmaceuticals-15-00008-s001.zip › pharmaceuticals-1499271-supplementary.pdf]

Repositioning of Etravirine as a potential CK1ε inhibitor by virtual screening

Luis Córdova-Bahena, Axel A. Sánchez-Álvarez, Angel J. Ruiz-Moreno, and Marco A. Velasco-Velázquez

Supplementary material

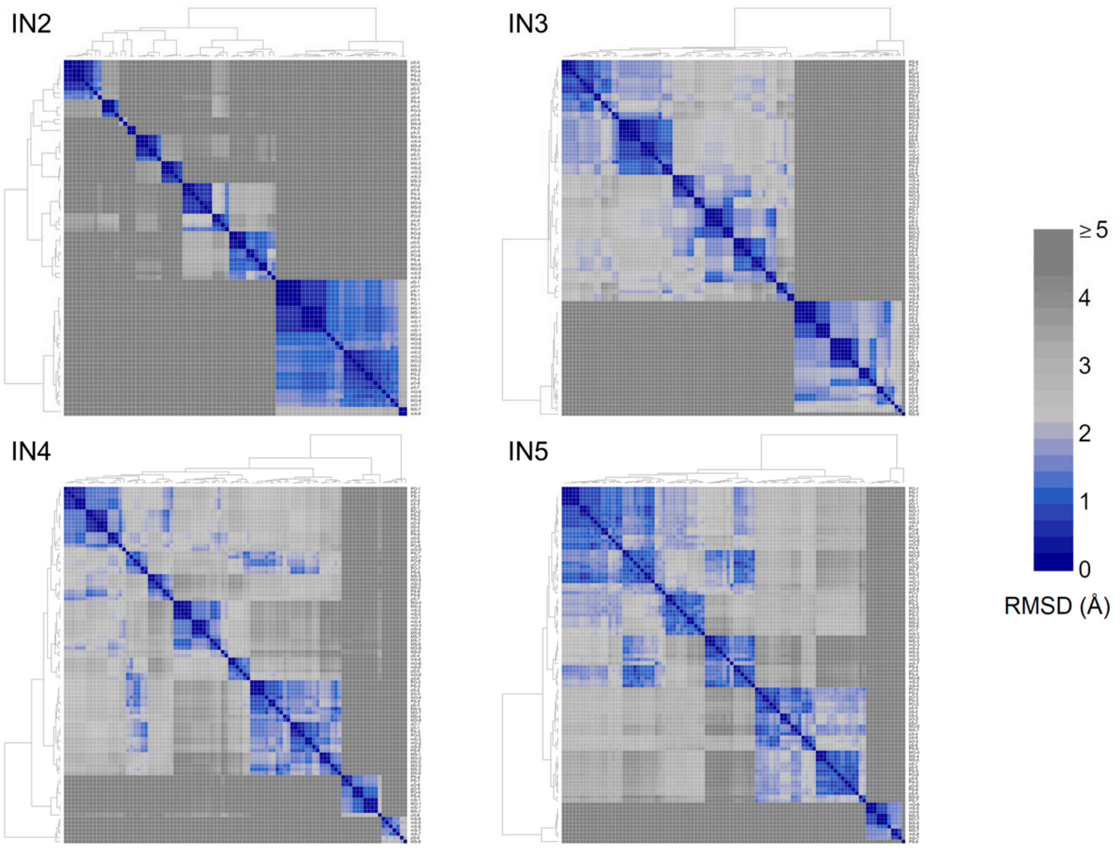

Figure S1. **Docking protocol for selected inhibitors set**. Hierarchical clustering of poses obtained by molecular docking. Color scale shows the RMSD between poses.

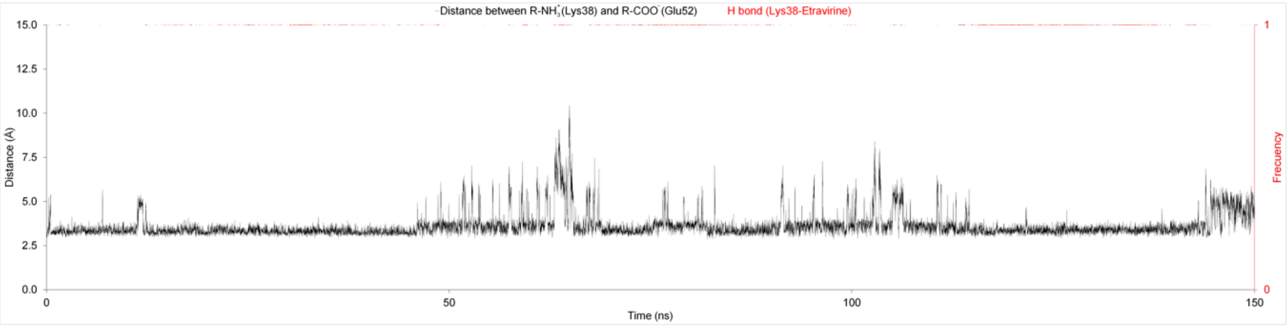

Figure S2. Salt bridge between Lys38 and Glu52 in CK1ε-Etravirine. Distance between the ammonium group of Lys38 and the carboxylate group of Glu52 on black line. Presence of hydrogen bond between Etravirine nitrile moiety and Glu52 during 150 ns MD indicated as red dots.

Table S1. Hits from virtual screening.

Drug name	Structure	Pharmacophore matching	Interest predominant specie at physiological pH	Binding mode in agreement with pharmacophore	Further studied
**Eravacycline**	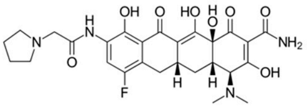	✓	✗	**N.D.**	✗
**Omadacycline**	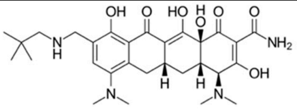	✓	✗	**N.D.**	✗
**Sarecycline**	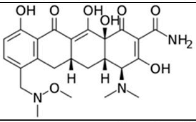	✓	✗	**N.D.**	✗
**Flupirtine**	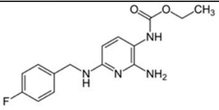	✓	✗	**N.D.**	✗
**Dolutegravir**	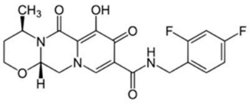	✓	✓	✗	✗
**Fostamatinib**	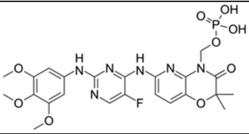	✓	✓	✗	✗
**Etravirine**	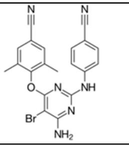	✓	✓	✓	✓
**Abacavir**	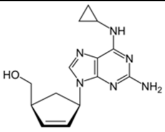	✓	✓	✓	✓

N.D.: not determined
